# Evaluation of Reflectivities of RPE, ELM, EZ, and Their Relationship With Subretinal Fluid Properties in Central Serous Chorioretinopathy

**DOI:** 10.1167/iovs.66.12.19

**Published:** 2025-09-08

**Authors:** Ekin Ece Oskan, Abdullah Ağın, Mine Ozturk, Feyza Onder

**Affiliations:** 1 Istanbul Aydın University, Department of Ophthalmology, Istanbul, Turkey; 2 University of Health Science, Haseki Training and Research Hospital, Department of Ophthalmology, Istanbul, Turkey

**Keywords:** central serous chorioretinopathy, reflectivity, external limiting membrane, ellipsoid zone, retinal pigment epithelium

## Abstract

**Purpose:**

The purpose of this study was to assess the reflectivity of the outer retinal layers (ORLs) in patients with central serous chorioretinopathy (CSCR) and to examine the relationship between the dimensions of the subretinal fluid (SRF) and ORL.

**Methods:**

This retrospective, cross-sectional study included 33 eyes of 33 patients with CSCR and 33 age- and gender-matched controls. Unnormalized and relative reflectivities for the retinal pigment epithelium (RPE), the external limiting membrane (ELM), and the ellipsoid zone (EZ), as well as SRF height, base width, and area, were measured on optical coherence tomography images. Reflectivity measurements for each retinal layer were performed at three anatomic locations (foveal center, nasal, and temporal regions, 1 mm apart), and the average of these three values was used to calculate average reflectivity (RPEav, EZav, ELMav).

**Results:**

RPEav, EZav, and ELMav were lower in patients with CSCR (*P* < 0.001). In the pigment epithelium detachment (PED) group, EZn and EZav were significantly lower than in the non-PED group (*P* = 0.012 and *P* = 0.013, respectively). A negative correlation was observed between SRF base width and EZav (*P* = 0.018) and ELMav (*P* = 0.021). SRF area was negatively correlated with both EZav (*P* = 0.049) and ELMav (*P* = 0.025). RPEc was negatively correlated with SRF elevation (*P* = 0.016).

**Conclusions:**

This study reveals novel associations between SRF dimensions, PED presence, and outer retinal layer damage in CSCR. Monitoring ORL reflectivity changes may provide insights into disease pathogenesis and help evaluate treatment efficacy.

Central serous chorioretinopathy (CSCR) is the fourth most common form of retinopathy, with a higher incidence observed in males.^1^ The typical lesion in CSCR is a well-circumscribed serous detachment, typically located in the macula.^2^ The pathogenesis is based on fluid leaking into the subretinal space, separating the retina from the underlying retinal pigment epithelium (RPE).^3^ Although the etiology of the disease remains incompletely understood, it is thought that increased choroidal capillary permeability and RPE dysfunction are the mechanisms responsible for the development of CSCR.

Patients with CSCR usually report a central scotoma and metamorphopsia in one eye. The differential diagnosis of CSCR includes conditions such as choroidal hemangioma, age-related macular degeneration, inflammatory retinopathies, polypoidal choroidal vasculopathy, macular holes or tears, optic disc pit maculopathy, other choroidopathies, and neoplastic lesions.^1^^,^^4^ Most patients recover spontaneously within 3 months. Degenerative changes in the outer retinal structures can cause permanent damage and poor final visual acuity in people who have had neurosensory detachment for a long time or more than once.

The primary pathology sites in CSCR are the choriocapillaris and outer retinal structures. CSCR is categorized as acute or chronic depending on the duration of subretinal fluid (SRF); eyes with SRF lasting over 3 to 6 months are characterized as having chronic CSCR.^5^ This time frame is derived from previous findings indicating that most new or recurrent instances of CSCR resolve within this period. Optical coherence tomography (OCT) is the gold-standard imaging technique for the diagnosis and follow-up of CSCR. The findings on OCT usually consist of neurosensory retinal detachment, pigment epithelium detachment (PED), cystic cavities, and fibrinous exudates. OCT offers reassurance about advances in medical technology with its potential as a diagnostic tool. Although OCT is a handy tool for assessment, it alone might not be sensitive enough to detect the submicroscopic changes in the outer retinal layers (ORLs), thereby requiring more sensitive quantitative methods. Literature on the quantitative assessment of ORL damage is scarce in patients with CSCR. For instance, Yucel Gencoglu et al.^6^ highlight the importance of measuring the reflectivities of RPE, ellipsoid zone (EZ), and external limiting membrane (ELM) in patients undergoing hydroxychloroquine treatment. The findings suggest that decreased reflectivity in these layers can serve as a predictive biomarker for retinal toxicity, thereby emphasizing the clinical relevance of OCT in monitoring treatment effects and preventing irreversible damage.^6^

Similarly, Hood et al.^7^ demonstrate that diminished cone function correlates with reduced intensity at the inner segment/outer segment (IS/OS) junction, suggesting that reflectivity changes can provide insight into the functional status of photoreceptors. This relationship between reflectivity and cone function underscores the potential of OCT as a diagnostic tool in assessing retinal health. Moreover, the work by Toprak et al.^8^ further illustrates how OCT can reveal alterations in reflectivity associated with idiopathic epimacular membranes. Their comparative study shows that changes in IS/OS junction reflectivity can indicate functional impairments in the retina, thus reinforcing the notion that OCT-derived reflectivity metrics are vital for understanding retinal pathologies and can provide a deeper understanding of the disease’s progression.^8^ Additionally, Murakami et al.^9^ discuss how OCT has transformed the assessment of diabetic macular edema, allowing for a more nuanced understanding of the disease’s pathophysiology beyond mere macular thickening.

As a fundamental aspect of retinal assessment, OCT offers valuable insights into the functional integrity of photoreceptors and the overall health of the retina. There are no data in the literature that assess the structural alterations in the outer retinal layers and their relationship with subretinal fluid, both morphologically and quantitatively. Evaluating the dimensions and reflectivity of the outer retinal layers in relation to the SRF in patients with CSCR could significantly advance the understanding of the pathophysiology and progression of the disease. Moreover, it may pave the way for personalized medical approaches, offering a promising avenue for future research and clinical practice.

## Patients and Methods

### Study Design and Ethics

The study was designed as a retrospective, cross-sectional observational analysis based on previously acquired high-resolution OCT images. Although retrospective in nature, the use of a standardized imaging protocol enhanced the reproducibility and interpretive consistency of the findings. Ethical approval from the Haseki Training and Research Hospital Ethics Committee was obtained before the commencement of the study (no. 138–2022). Written informed consent was waived due to the retrospective nature of the study, and the study was conducted in accordance with the ethical principles set forth in the Declaration of Helsinki.

### Study Population

The patient group comprised 21 males (63.6%) and 12 females (36.4%), whereas the control group included 19 males (57.6%) and 14 females (42.4%). The mean age of the patient group was 45.5 ± 11.5 years (range: 26–66 years), similar to the control group, which had a mean age of 44.3 ± 11.7 years (range: 21–62 years). Visual acuity in the patient group, expressed as LogMAR, was 0.31 ± 0.24 (range: 0–1), compared to 0 ± 0.00 in the control group. The symptom duration for patients was 7.8 ± 9.4 months (range: 0.1–28 months). Among the patients, 20 (60.6%) were categorized as acute, while 13 (39.4%) were chronic cases. The presence of PED was noted in 5 patients (15.2%), whereas 28 (84.8%) had no PED. Eligibility criteria for the patient group included being over 18 years of age and having no ocular pathology that could affect the retina other than CSCR. Individuals with inflammatory eye disease, a history of retinal surgery, a history of cataract surgery within the past 6 months, a refractive error greater than ± 3 diopter, ocular media opacities that could affect image quality, or a scan quality index (SQI) of less than 7/10 were not eligible. Patients with other posterior segment pathologies that could structurally affect the retina, such as senile macular degeneration, diabetic retinopathy, retinal vascular occlusion, glaucoma, and degenerative myopia, were also ineligible.

The diagnosis of CSCR was based on a comprehensive ophthalmic examination, including fundoscopy, OCT, and fundus fluorescein angiography. Demographic data, such as age, sex, systemic diseases, medications, subjective visual complaints, and duration of symptoms, were also recorded. As part of the exam, the patient’s best-corrected visual acuity, slit-lamp biomicroscopy of the anterior segment, intraocular pressure measurement, fundoscopy, and OCT evaluation were all checked.

### Imaging and Measurement Protocol

High-resolution cross-sectional B-scan OCT images were obtained for both the CSCR and control groups using the AngioVue Avanti RTVue-XR device (Optovue, Fremont, CA, USA). In vivo imaging was performed on 33 eyes of 33 patients during either acute CSCR or an acute exacerbation of chronic CSCR with SRF. All images were acquired under pharmacologic mydriasis using the HD Line protocol. For each patient, a single horizontal B-scan centered on the fovea was used for analysis. Each scan consisted of 1024 A-scans across a 6-mm horizontal field of view, with 25-frame averaging applied to enhance image quality and reduce speckle noise. The device's axial and lateral resolutions were 5 µm and 15 µm, respectively. All scans were obtained by the same experienced operator (M.O.) under standardized fixation and alignment conditions. Only scans with an SQI ≥7/10 were included to ensure an adequate signal-to-noise ratio and minimize measurement variability. Notably, no variations in frame averaging, scan width, or device settings occurred across patients.

### Reflectivity Analysis

The acquired images were subsequently imported into ImageJ software (version 1.8.0_77; National Institutes of Health, Bethesda, MD, USA) for reflectivity analysis. This is an open-access, Java-based image-processing program that enables the user to transform specific image characteristics into quantitative data. The consistency of measurements between users and by the same user proves that the software is a widely adopted, validated image analysis tool in ophthalmic research, with documented reproducibility in prior studies.^10^^–^^12^ The height and base width of the SRF in the CSCR group were measured using the “straight-line” function of the ImageJ software. The area of the SRF was calculated using “segmented lines,” and quantitative data were obtained.

A vertical straight line was drawn in the vitreous-choroid direction from the foveal center, perpendicular to the RPE, and the points 500 microns away from the foveal center in the nasal and temporal directions. The unnormalized reflectivities of the RPE, EZ, and ELM were calculated for each of the three vertical straight lines, 500 microns apart from each other (central [c], temporal [t], and nasal [n]), using the “plot profile” function of the software. A reflectivity graph was obtained for each point.

Reflectivity measurements were manually performed at three anatomic locations: central (foveal center), nasal, and temporal. The central point was identified as the location of maximum SRF accumulation or the anatomic foveal depression, if visible. The nasal and temporal measurements were placed approximately 1 mm from the foveal center along the horizontal meridian. The average reflectivity (RPEav, EZav, ELMav) for each layer was calculated as the mean of the three measurements (central, nasal, temporal). At each location, a vertical segment encompassing five to seven adjacent A-scans was selected, positioned perpendicular to the RPE curvature to minimize directional reflectivity artifacts. All measurements were conducted using ImageJ software. Two independent graders (E.E.O. and A.A.), masked to the clinical diagnosis, performed the measurements. Interobserver agreement was assessed in a randomly selected subset of 20 eyes. All measurement points, including the nasal and temporal locations, were placed within areas elevated by SRF to ensure consistent fluid exposure across all sampled regions.

**Figure 1.**
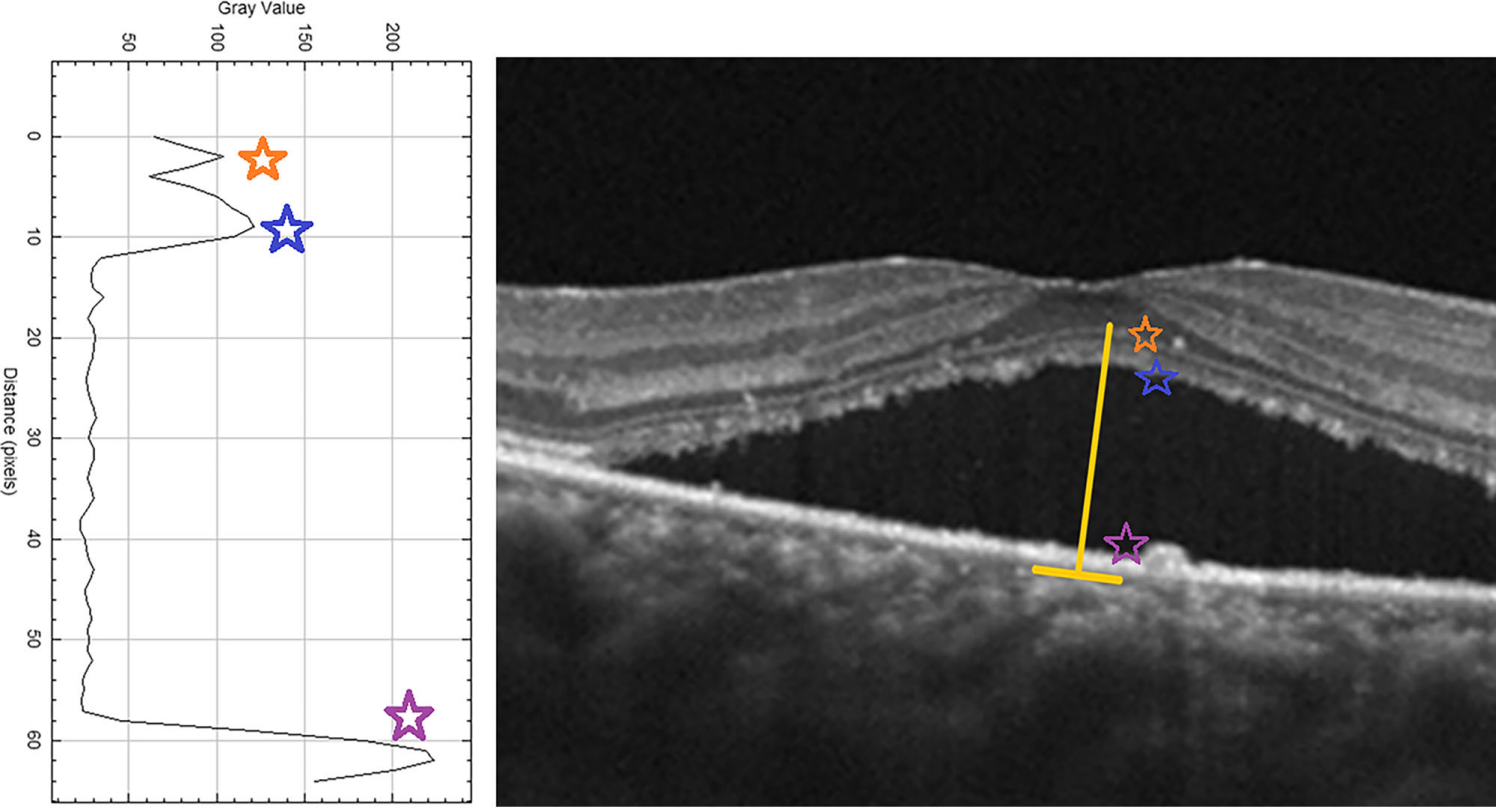
On the right side, the line where the central reflectivity is measured in the cross-sectional OCT (*yellow line*) is shown, and the RPE (*purple star*), EZ (*blue star*), and ELM (*orange star*) layers are shown from outside to inside. Each vertical line segment used for reflectivity measurement spans approximately five to seven adjacent A-scans and is positioned perpendicular to the local curvature of the RPE to reduce directional artifacts.

**Figure 2.**
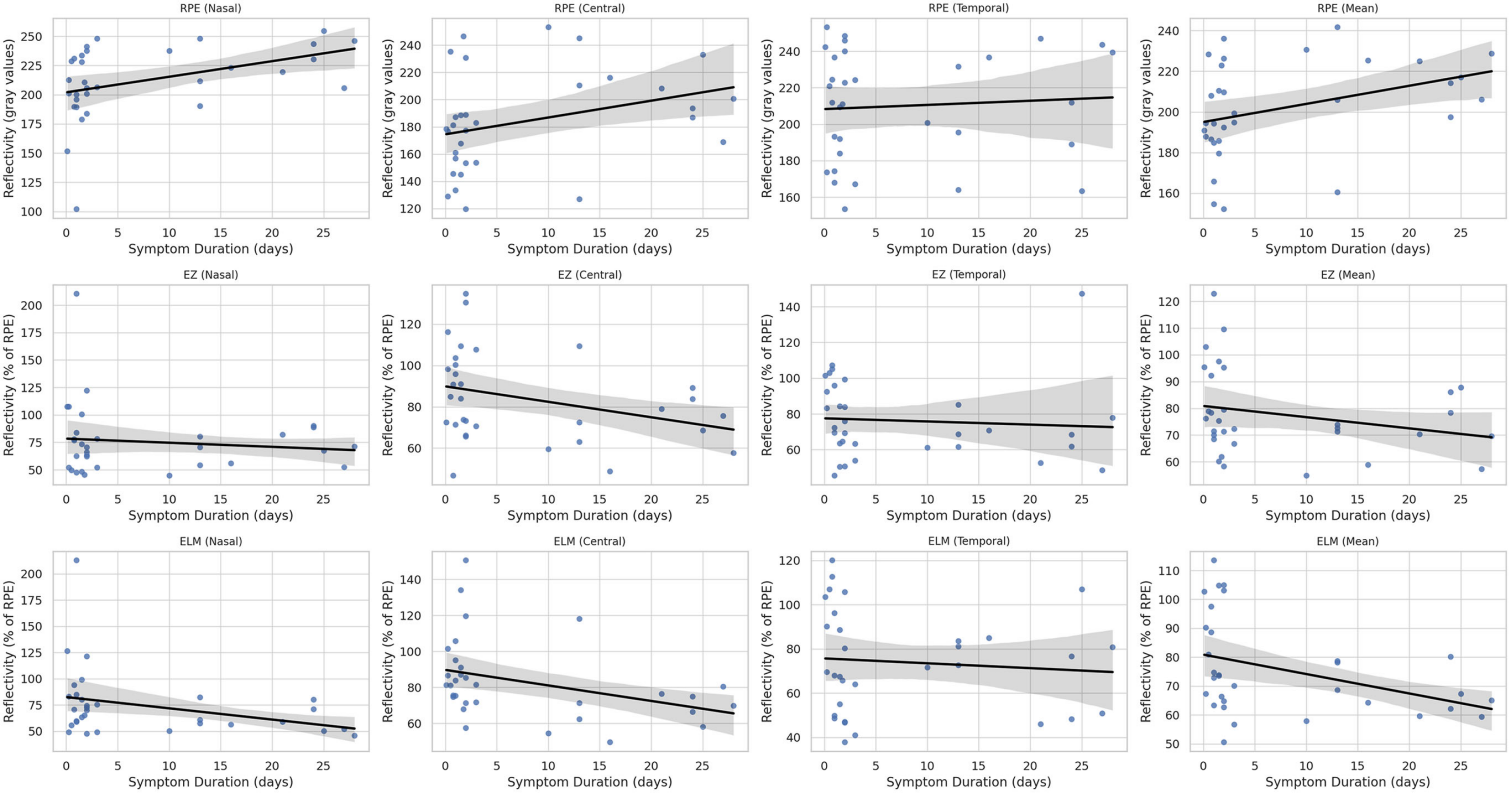
Scatterplots showing the correlation between symptom duration and the unnormalized reflectivity values of three outer retinal layers, RPE, EZ, and ELM, at four anatomic locations: nasal, central, temporal, and the average of these points. All reflectivity values are presented in grayscale intensity units (gray values). Each point represents a single patient. Linear regression trendlines with 95% confidence intervals are included for reference.

**Figure 3.**
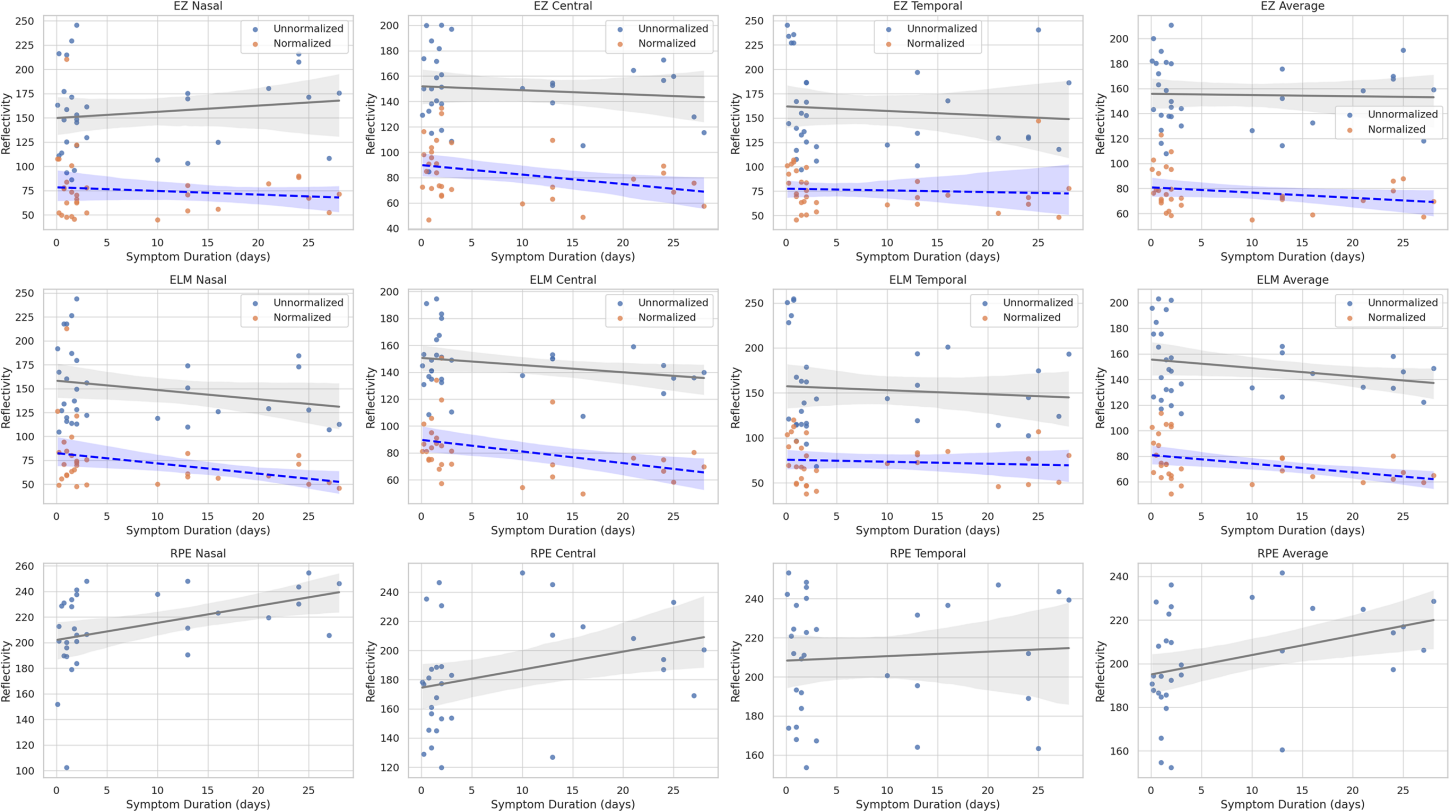
Composite scatterplot illustrating the correlation between symptom duration and both unnormalized (gray values) and normalized (relative) (% of RPE reflectance) reflectivity values of the outer retinal layers. Different markers and colors distinguish the EZ, ELM, and RPE across normalized and raw reflectance scales.

**Figure 4.**
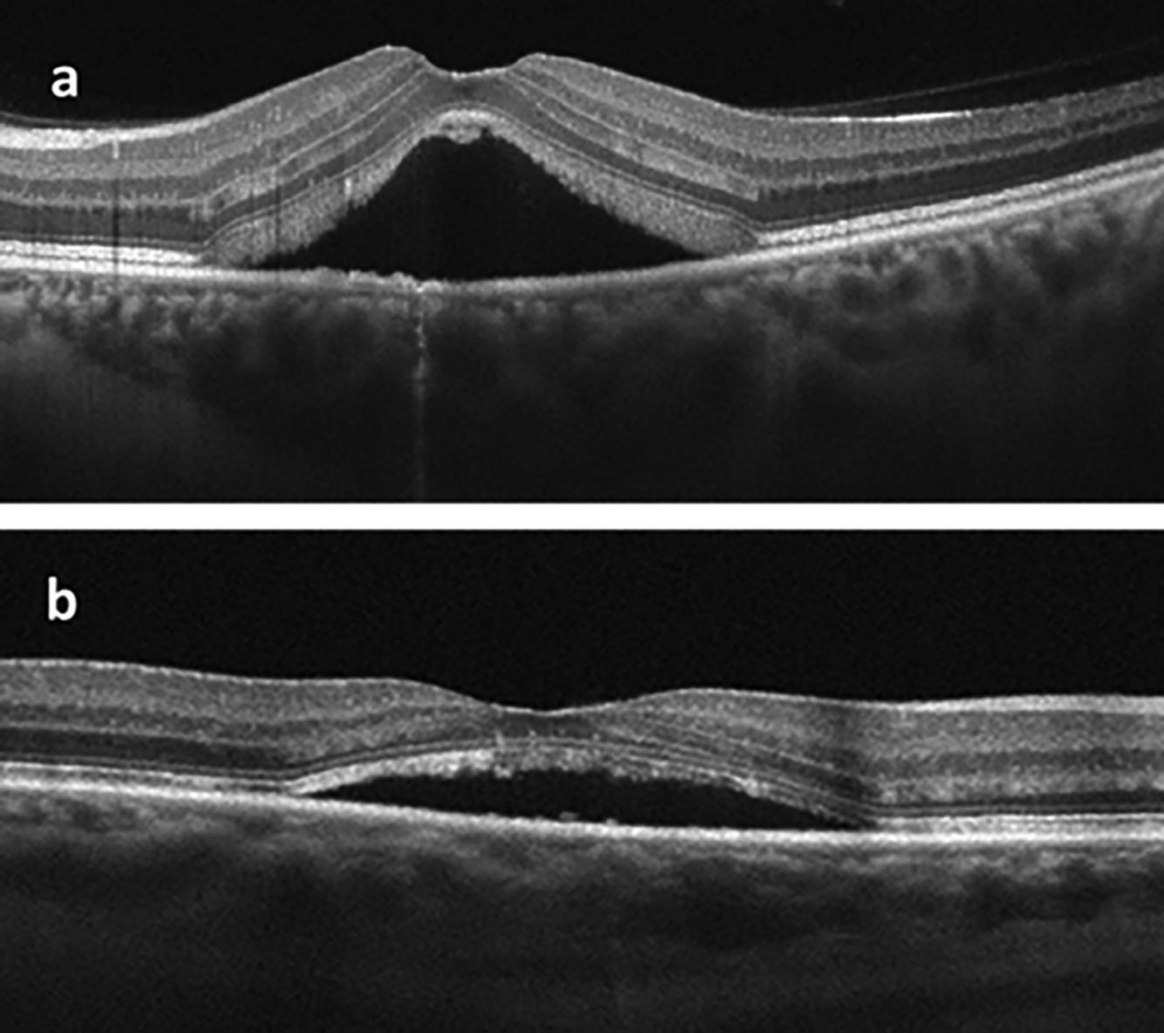
Representative B-scan OCT images from two CSCR eyes showing variations in outer retinal reflectivity. (**a**) A case with pronounced attenuation of the EZ and ELM reflectivity, accompanied by prominent subretinal fluid. (**b**) A case with relatively preserved outer retinal reflectivity despite the presence of a lesser amount of subretinal fluid. Quantitative measurements corresponding to these structural changes are described in Figure 1.

In the cross-sectional OCT image of a healthy adult, when the hyperreflective bands of the outer retina are evaluated histologically, the order of reflectivity from highest to lowest is as follows: RPE, EZ (also called photoreceptor IS/OS band), and ELM. The outermost layer with the highest reflectivity represents the RPE band. The relative reflectivities of the EZ and ELM bands were calculated from the unnormalized reflectivity of the RPE band. The relative reflectivity was calculated by dividing the unnormalized reflectivities of the EZ and ELM bands by the unnormalized reflectivity of the RPE band and multiplying the result by 100.^10^^–^^12^ In Figure 1, the measurement technique was shown in both the OCT cross-sectional image and the ImageJ program. To enhance interpretability and facilitate direct comparison, we created a set of scatterplots that illustrate both unnormalized and relative (RPE-normalized) reflectivity values for each outer retinal layer (Fig. 2). Distinct colors and marker styles were used to distinguish unnormalized values (in gray values) from normalized (relative) reflectivity values (expressed as a percentage of RPE reflectance). To further investigate whether changes in RPE reflectivity influenced the observed trends in normalized RelEZ and RelELM reflectivity, we also generated a composite figure that overlays raw and normalized data in a single panel (Fig. 3). The persistence of a negative correlation in the raw reflectivity values supports the presence of accurate signal attenuation in the EZ and ELM, independent of normalization to RPE. Finally, to provide visual context for the range of structural changes observed, representative B-scan OCT images from two CSCR eyes with differing degrees of outer retinal reflectivity attenuation are presented in Figure 4.

### Statistical Analysis

Statistical analyses were performed using the SPSS 22.0 software package (Statistical Package for Social Sciences; SPSS Inc., IBM, Armonk, NY, USA). Descriptive analyses were presented using proportions, medians, and minimum and maximum values, as appropriate. The conformity of the variables to a normal distribution was investigated using visual (histograms, probability plots) and analytical methods (Kolmogorov–Smirnov). Comparisons of numerical variables between two independent groups were analyzed using the Student’s *t*-test for the normal distribution and the Mann–Whitney *U* test for the nonnormal distribution. Linear regression analysis was performed to adjust for the effect of age between the acute and chronic CSCR groups, which had a significant age difference. The relationship between continuous variables was analyzed using Pearson's correlation coefficient when both variables met the assumptions of normal distribution and linearity. For variables with nonnormal or skewed distributions, such as symptom duration, Spearman's rank correlation was used to assess monotonic relationships without assuming parametric conditions. Interobserver agreement was assessed using intraclass correlation coefficients (ICCs). The alpha significance level was accepted as *P* ˂ 0.05.

## Results

### Reflectivity Characteristics of the Patient and Control Groups

As shown in Table 1, the patient group exhibited significantly lower reflectivity values for RPEc (184.6 ± 36.6), EZc (149.7 ± 27.9), and ELMc (147.5 ± 21.5) compared to the control group, where these values were 231.9 ± 11.5, 223.4 ± 18.8, and 188.9 ± 29.5, respectively (*P* < 0.001 for all). Similar reductions were observed for EZt, ELMt, EZn, and ELMn in the patient group (*P* < 0.001). In contrast, RPEn values showed no significant difference between groups (*P* = 0.267). These findings indicate substantial structural alterations in the retinal layers among patients.

**Table 1.** RPE, EZ, and ELM Reflectivities in the CSCR Patient and Control Groups

	CSCR Group		Control Group		
Characteristic	Mean ± SD	Min–Max (Median)	Mean ± SD	Min–Max (Median)	*P* Value
RPEc	184.6 ± 36.6	119.7–253.2 (183)	231.9 ± 11.5	202.1–247.8 (233.8)	<0.001^*^
RPEn	212.4 ± 30.9	102.3–254.5 (212.8)	221.5 ± 12.0	197.6–243.2 (217.5)	0.267^*^
RPEt	209.3 ± 30.0	153.6–253.2 (212)	219.0 ± 12.9	195.6–243.6 (217.9)	0.097^†^
RPEav	201.9 ± 23.3	152.3–241.6 (205.8)	224.1 ± 9.5	204.6–240.9 (223.2)	<0.001^*^
EZc	149.7 ± 27.9	84.7–200.2 (151.3)	223.4 ± 18.8	181–254.3 (225.2)	<0.001^*^
EZn	154.4 ± 42.3	86.4–245.8 (153.2)	225.3 ± 16.3	189.7–250.9 (231.3)	<0.001^*^
EZt	157.7 ± 44.9	96.9–245.5 (139.8)	230.4 ± 15.2	193.3–250.8 (229.6)	<0.001^*^
EZav	154.7 ± 26.4	108–210.9 (152.3)	226.4 ± 8.4	204.3–241.7 (228.3)	<0.001^*^
ELMc	147.5 ± 21.5	107.4–194.5 (145.1)	188.9 ± 29.5	145–246.1 (179.2)	<0.001^†^
ELMn	150.4 ± 38.3	104.8–243.8 (135.7)	227.9 ± 16.5	189.0–250.8 (231.2)	<0.001^†^
ELMt	154.0 ± 49.7	68.5–254.5 (143.7)	230.7 ± 15.4	194.7–253.3 (233.7)	<0.001^*^
ELMav	150.7 ± 25.4	113.3–203 (146.7)	215.8 ± 11.4	195.6–246 (215.6)	<0.001^*^
RelEZc	83.9 ± 21.7	46.8–134.7 (79)	96.3 ± 6.8	83.9–107 (97.5)	0.003^*^
RelEZn	75.3 ± 31.1	44.9–210.3 (70.6)	102.0 ± 9.1	81.5–118.2 (100.6)	<0.001^*^
RelEZt	76.0 ± 21.7	45.5–147.2 (70.8)	105.6 ± 9.2	85.5–126.2 (105.9)	<0.001^*^
RelEZav	77.5 ± 15.8	54.9–122.9 (72.7)	101.2 ± 5.5	91.0–113.5 (101.4)	<0.001^*^
RelELMc	83.2 ± 22.3	49.7–150.6 (80.4)	81.4 ± 11.7	63.5–109 (81.9)	0.773^*^
RelELMn	74.0 ± 31.7	45.8–213 (65.1)	103.3 ± 10.2	78.7–119.3 (102.9)	<0.001^*^
RelELMt	74.2 ± 22.9	38–120.1 (72.8)	105.8 ± 10.9	84.5–123.8 (105.7)	<0.001^*^
RelELMav	75.8 ± 16.4	50.7–113.5 (72.9)	96.6 ± 7.8	81.2–112.2 (96.5)	<0.001^*^

* Mann–Whitney *U* test.

† Student's *t*-test.

Reflectivity changes and clinical features in the acute and chronic groups with age adjustment using linear regression analysis are detailed in Table 2. The acute CSCR group had a significantly lower mean age (41.0 ± 9.6 years) compared to the chronic group (52.3 ± 11.1 years, *P* = 0.004). The duration of symptoms was notably shorter in the acute CSCR group (1.3 ± 0.8 months) versus the chronic CSCR group (17.8 ± 7.6 months, *P* < 0.001). In the acute CSCR group, several differences are observed compared to the chronic group. RPEc was significantly lower in the acute group (*P* = 0.028), as well as RPEn (*P* = 0.018) and RPEav (*P* = 0.020). On the other hand, EZt was higher in the acute group (*P* = 0.009), as well as ELMt (*P* = 0.006) and ELMn (*P* = 0.018). Additionally, EZav (*P* = 0.017) and ELMav (*P* = 0.010) were also higher in the acute group, as determined by age-adjusted linear regression analysis. Besides, all parameters of relative reflectivity were higher in the acute CSCR group than in the chronic group, as determined in the same way by age-adjusted linear regression analysis. These findings suggest that while RPE reflectivity values were generally lower in the acute group, EZ and ELM bands demonstrated relatively higher reflectivity signals. It is important to note that increased reflectivity of the EZ and ELM bands does not necessarily indicate complete structural preservation. Reflectivity values should be interpreted as indirect markers of optical and microstructural changes, not as definitive indicators of anatomic integrity. The three outer retinal layers evaluated in this study differ in both physiological and morphological properties: the RPE is a melanin-rich epithelial monolayer with inherently high reflectance, the EZ corresponds to the mitochondria-dense ellipsoid portion of photoreceptors, and the ELM is a thin junctional line formed by Müller cell photoreceptor interfaces. Therefore, changes in reflectivity may reflect different pathophysiological mechanisms affecting these layers in CSCR, rather than uniform structural damage or preservation. As highlighted in Table 3, significant differences were observed in reflectivity values between groups with and without PED. Patients with PED showed lower EZn values (111.9 ± 19.4) compared to those without PED (162.0 ± 40.8, *P* = 0.012), and EZav values were also significantly lower in the PED group (128.2 ± 12.7 vs. 159.5 ± 25.5, *P* = 0.013). However, no statistically significant differences were observed in RPEc or ELMav values between the two groups (*P* > 0.05). Additionally, measurements for SRF base, height, and area showed no significant variations between the PED and non-PED groups (*P* > 0.05). These findings emphasize the distinct reflectivity and structural changes in patients with PED. Interobserver agreement (E.E.O., A.A.) for all reflectivity parameters was excellent, with ICCs ranging from 0.91 to 0.97 across all anatomic locations.

**Table 2.** Examination Findings in Acute and Chronic CSCR Groups: RPE, EZ, and ELM Reflectivities

	Acute CSCR Group		Chronic CSCR Group				
Characteristic	Mean ± SD	Min–Max (Median)	Mean ± SD	Min–Max (Median)	*P*	*P* ^*^	*P* ^†^
Age	41.0 ± 9.6	26–62 (40)	52.3 ± 11.1	31–66 (51)	0.004^‡^		
BCVA (logMAR)	0.30 ± 0.22	0.04–1 (0.3)	0.33 ± 0.28	0–1 (0.22)	0.794^§^		
Symptom duration	1.3 ± 0.8	0–3 (1.25)	17.8 ± 7.6	3–28 (16)	<0.001^§^		
RPEc	174.3 ± 34.2	119.7–246.5 (119.7)	200.5 ± 35.5	127–253.2 (208.3)	0.042^‡^	0.157	0.028
EZc	154.0 ± 31.0	84.7–200.2 (84.7)	143.2 ± 22.0	105.4–172.8 (152.6)	0.284^‡^	0.342	0.284
ELMc	152.1 ± 22.2	108.6–194.5 (108.6)	140.5 ± 19.2	107.4–178.1 (139.9)	0.133^‡^	0.158	0.542
RPEt	208.7 ± 31.1	153.6–253.2 (153.6)	210.4 ± 29.3	163.4–247 (212)	0.877^‡^	0.935	0.243
EZt	164.5 ± 47.8	96.9–245.5 (96.9)	147.1 ± 39.5	101–240.6 (130.8)	0.298^§^	0.667	0.009
ELMt	155.9 ± 59.1	68.5–254.5 (68.5)	151.0 ± 32.1	102.5–201.1 (145)	0.768^§^	0.639	0.006
RPEn	203.6 ± 34.0	102.3–248.1 (102.3)	226.0 ± 19.4	190.5–254.5 (223.2)	0.033^§^	0.302	0.018
EZn	152.5 ± 46.1	86.4–245.8 (86.4)	157.4 ± 37.2	103.3–216 (169.9)	0.750^‡^	0.803	0.513
ELMn	157.9 ± 43.5	104.8–243.8 (104.8)	139.0 ± 26.2	107.1–184.5 (129.2)	0.253^§^	0.658	0.018
RPEav	195.5 ± 23.0	152.3–236.1 (152.3)	211.8 ± 20.7	160.5–241.7 (214.2)	0.044^‡^	0.183	0.020
Ezav	158.3 ± 28.1	108–210.9 (108)	149.2 ± 23.7	114.4–190.7 (152.3)	0.343^‡^	0.654	0.017
ELMav	155.5 ± 30.1	113.3–203 (113.3)	143.5 ± 13.7	122.4–165.9 (144.9)	0.133^‡^	0.600	0.010
RelEZc	90.8 ± 22.6	46.8–134.7 (46.8)	73.1 ± 15.5	48.7–109.4 (72.5)	0.019^‡^	0.091	0.028
RelELMc	90.3 ± 22.7	57.4–150.6 (57.4)	72.2 ± 17.1	49.7–118.1 (71.3)	0.005^§^	0.357	0.028
RelEZt	79.0 ± 19.4	45.5–107.1 (45.5)	71.5 ± 25.0	48.4–147.2 (68.5)	0.113^§^	0.740	0.008
RelELMt	75.1 ± 26.3	38–120.1 (38)	72.9 ± 17.1	46.1–106.9 (76.7)	0.773^‡^	0.628	0.005
RelEZn	79.1 ± 38.1	45.5–210.3 (45.5)	69.4 ± 14.4	44.9–90.1 (70.7)	0.971^§^	0.740	0.019
RelELMn	82.0 ± 37.8	47.6–213 (47.6)	61.7 ± 11.9	45.8–82.2 (58.9)	0.071^§^	0.556	0.016
RelEZav	81.9 ± 17.4	58.3–122.9 (58.3)	70.6 ± 10.0	54.9–87.9 (70.4)	0.039^§^	0.234	0.013
RelELMav	80.7 ± 18.6	50.7–113.5 (50.7)	68.2 ± 7.8	57.9–80.2 (67.3)	0.077^§^	0.237	0.011

BCVA, best-corrected visual acuity.

* Age-removed *P* value.

† Age-adjusted *P* value using linear regression analysis.

‡ Student t test.

§ Mann–Whitney *U* test.

**Table 3.** Examination Findings, SRF Characteristics, and RPE, EZ, and ELM Reflectivities in Groups With and Without PED

	PED				
	Positive		Negative		
Characteristic	Mean ± SD	Min–Max (Median)	Mean ± SD	Min-Max (Median)	*P*
Age	43.4 ± 14.7	31–66 (38)	45.8 ± 11.1	26–65 (45)	0.671^*^
BCVA (logMAR)	0.32 ± 0.23	0–0.52 (0.4)	0.31 ± 0.25	0.04–1 (0.26)	0.648^†^
Symptom duration	13.2 ± 9.1	1.8–27 (13)	6.8 ± 9.3	0.1–28 (2)	0.096^†^
RPEc	201.0 ± 53.3	127–253.2 (209.1)	181.7 ± 33.2	119.7–245.3 (182.1)	0.285^*^
EZc	150.4 ± 20.2	128–181.8 (150.5)	149.6 ± 29.4	84.7–200.2 (152)	0.952^*^
ELMc	153.8 ± 18.5	136–178.1 (150)	146.4 ± 22.1	107.4–194.5 (145)	0.486^*^
RPEt	201.4 ± 29.5	164–243.7 (200.7)	210.8 ± 30.3	153.6–253.2 (216.5)	0.526^*^
EZt	122.4 ± 14.2	101–136.1 (122.5)	164.0 ± 45.7	96.9–245.5 (153.8)	0.050^†^
ELMt	135.1 ± 12.8	119.3–149.3 (139)	157.3 ± 53.1	68.5–254.5 (151.9)	0.616^†^
RPEn	213.2 ± 17.6	190.5–237.7 (210.8)	212.3 ± 32.9	102.3–254.5 (216.1)	0.802^†^
EZn	111.9 ± 19.4	95.9–145.5 (106.7)	162.0 ± 40.8	86.4–245.8 (162.3)	0.012^*^
ELMn	121.8 ± 14.1	107.1–137.1 (119.2)	155.5 ± 39.2	104.8–243.8 (150.3)	0.063^†^
RPEav	205.2 ± 27.2	160.5–230.5 (206.2)	201.3 ± 23.0	152.3–241.7 (198.4)	0.740^*^
EZav	128.2 ± 12.7	114.4–144.2 (126.6)	159.5 ± 25.5	108–210.9 (159)	0.013^*^
ELMav	136.9 ± 13.7	122.4–154.4 (133.6)	153.2 ± 26.3	113.3–203 (147.7)	0.190^*^
RelEZc	78.3 ± 18.6	59.4–109.4 (73.8)	84.9 ± 22.4	46.8–134.7 (83.8)	0.541^*^
RelELMc	81.2 ± 23.8	54.4–118.1 (80.4)	83.6 ± 22.4	49.7–150.6 (78.8)	0.725^†^
RelEZt	61.4 ± 8.4	48.4–71.6 (61.6)	78.6 ± 22.4	45.5–147.2 (74.1)	0.056^†^
RelELMt	68.2 ± 10.8	50.9–79.7 (71.6)	75.3 ± 24.4	38–120.1 (78.5)	0.304^*^
RelEZn	52.6 ± 8.5	44.9–65.8 (52.6)	79.3 ± 31.9	47.7–210.3 (72.4)	0.010^†^
RelELMn	57.3 ± 6.2	50.1–65.1 (57.7)	77.0 ± 33.5	45.8–213 (70.9)	0.120^†^
RelEZav	63.1 ± 7.4	54.9–71.3 (61.9)	80.1 ± 15.5	58.3–122.9 (75.8)	0.008^†^
RelELMav	67.5 ± 9.3	57.9–78.8 (66.3)	77.2 ± 17.0	50.7–113.5 (73.2)	0.269^†^
SRF base	1423.3 ± 525.6	1028.7–2280.6 (1136.8)	1053.5 ± 365.6	231.6–1899.7 (1099.3)	0.060^*^
SRF height	68.3 ± 18.1	42.1–92.2 (68.1)	84.6 ± 66.7	15.1–314.5 (69)	0.841^†^
SRF area	17,126.0 ± 3158.7	13,060.9–21,350.4 (16,392)	13,230.9 ± 4934.7	2818.6–23,067.9 (13,836.6)	0.101^*^

* Student's *t*-test.

† Mann–Whitney *U* test.

### SRF Characteristics in Acute and Chronic Groups

The SRF base width in the acute CSCR group was 1047.7 ± 337.3 µm (range: 398.7–1576.6 µm), while in the chronic group, it was 1204.6 ± 494.7 µm (range: 231.6–2280.6 µm). After age adjustment using linear regression analysis, this difference was statistically significant (*P* = 0.01). The SRF height was 95.0 ± 74.2 µm (range: 15.1–314.5 µm) in the acute group and 62.4 ± 28.6 µm (range: 15.3–100.6 µm) in the chronic group. The age-adjusted analysis indicated a significant difference with *P* = 0.025. The SRF area was 13,005.2 ± 4726.0 µm² (range: 4626–21,488.9 µm²) in the acute CSCR group and 15,076.2 ± 5029.8 µm² (range: 2818.6–23,067.9 µm²) in the chronic CSCR group, again showing no significant difference (*P* = 0.372, age-adjusted linear regression analysis).

### Correlation Analyses

Statistically significant correlations were observed in the following parameters: a moderate negative correlation between SRF height and RPEc (*r* = −0.415, *P* = 0.016). Symptom duration showed a positive correlation with RPEc (*r* = 0.379, *P* = 0.030). SRF width exhibited a significant negative correlation with EZt (*r* = −0.410, *P* = 0.018) and ELMt (*r* = −0.401, *P* = 0.021). SRF area correlated negatively with EZt (*r* = −0.359, *P* = 0.040) and ELMt (*r* = −0.347, *P* = 0.048). No significant correlations were identified for other metrics.

## Discussion

CSCR is a retinal disease characterized by serous detachment of the neurosensory retina, with changes typically confined to the macula and associated with fluid leakage from the RPE into the subretinal space. SRF, a vital part of CSCR, builds up under the retina as the pumping function of the RPE fails, which is an essential part of how the disease starts. The reflectivities of the RPEc, EZc, ELMc, EZt, ELMt, EZn, ELMn, RPEav, EZav, and ELMav were lower than that of the control group (*P* < 0.001). This process results in the separation of the neurosensory retina from the underlying RPE layer. As photoreceptors are separated from the RPE, they undergo degenerative changes over time, resulting in deterioration of the EZ and ELM layers, which are components of the outer retina. So, even though choriocapillaris is where the primary damage occurs in CSCR, the disease gets worse as the RPE, EZ, and ELM break down. On the other hand, the dimensional characteristics that reflect the amount of SRF and the degree of damage to the outer retinal structures have yet to be fully elucidated in the literature to date, and there are minimal data in the literature on these patients.

The width, area, and elevation of SRF demonstrate some negative correlations with the reflectivity values of the outer retinal layers. As the SRF base width and area increase, EZav and ELMav decrease significantly (*P* < 0.05). Similarly, increased SRF elevation is associated with a reduction in the reflectivity of RPEc (*P* = 0.016). It is plausible that prolonged exposure to SRF interferes with the physiological environment of the retina, which could partially explain the observed changes in reflectivity. A study conducted by Cong et al.^13^ explored the relationship between the degree of disintegrity of the EZ and the width and height of SRF. However, the results did not indicate a significant correlation between the disintegrity of the EZ and the width or height of the SRF.

On the other hand, in this study, disintegration of EZ was classified as follows: 1, 100% disintegrity; 2, 50% to 100% disintegrity; 3, 0% to 50% disintegrity; and 4, 0% disintegrity.^13^ In our study, reflectance alterations in the EZ and ELM bands were more clearly detected using the current protocol, and these changes were assessed more accurately through quantitative analysis. Furthermore, not only the presence of disintegration but also the reflectivity, which provides insight into the functional integrity of these structures, was analyzed in three layers of the outer retina.

### Reflectivity Changes and Outer Retinal Layer Integrity

The significant reduction in reflectivities of the RPE, EZ, and ELM in patients with CSCR compared to controls points to extensive structural damage within the outer retinal layers, especially in the presence of SRF. This reduction in reflectivity is in line with the findings of recent studies investigating the integrity of outer retinal structures in CSCR. Maltsev et al.^14^ demonstrated that photoreceptor outer segment (PROS) thinning is closely associated with visual prognosis, particularly in acute CSCR cases, and is highly correlated with the reflectivity measurements of the EZ layer in OCT scans. The thinning of the EZ layer overlying SRF or leakage areas may indicate photoreceptor damage, which can be quantitatively evaluated through the reflectivity metrics applied in the present study.

Fujita et al.^15^ emphasized the importance of quantifying residual EZ to evaluate visual outcomes in patients with resolved CSCR. Their study found that higher residual EZ values correlated with better visual acuity, suggesting that preserving EZ integrity is critical for visual recovery. This is consistent with our findings that decreased reflectivity of the EZ is associated with higher SRF sizes and the presence of PED. This suggests that SRF leads to reduced integrity of the photoreceptor elements, subsequently impairing visual function.^15^ The chronic CSCR group demonstrates higher RPE reflectivity metrics compared to the acute group, particularly in age-adjusted analyses. Significant differences in RPEn (*P* = 0.018) and RPEav (*P* = 0.020) suggest cumulative damage or compensatory changes in RPE over time, which may be associated with prolonged disease activity and chronicity. EZ and ELM reflectivities also differ between acute and chronic CSCR groups, with the chronic group CSCR generally showing lower values after age adjustment (e.g., EZav, *P* = 0.017; ELMav, *P* = 0.010). These findings indicate photoreceptor and ORL degeneration in chronic cases, likely due to sustained damage from persistent SRF or other pathological mechanisms inherent to chronic CSCR. In addition, the relative reflectivity values showing the rate of EZ or ELM to RPE were observed to be higher in the acute CSCR group compared to the chronic CSCR group in age-adjusted linear regression analysis. These two findings strongly support each other. While we observed consistent attenuation of reflectivity in certain outer retinal bands, we acknowledge that these findings represent optical signal changes rather than definitive signs of structural disintegration. In the absence of apparent morphological alterations such as layer thinning or fragmentation, reflectivity changes should be interpreted cautiously, as possible early indicators of microstructural stress. The duration of symptoms may serve as a surrogate marker for the cumulative exposure of the outer retina to SRF. Prolonged symptom duration could lead to chronic photoreceptor stress or metabolic disruption, ultimately contributing to reduced reflectivity in the EZ and ELM bands. This interpretation is supported by the observed inverse correlation between symptom duration and reflectivity values in Figures 2 and 3, suggesting a time-dependent degenerative process in CSCR.

### Impact of SRF Dimensions on Retinal Structures

The negative correlation between the SRF area and the mean reflectivity of both the EZ and ELM emphasizes the impact of SRF dimensions on the outer retina’s structural integrity. Cong et al.^13^ investigated the relationship between SRF absorption and structural integrity of retinal layers, finding that larger SRF volumes were associated with increased retinal disorganization and longer recovery times. In the current study, SRF base width and area were both inversely correlated with reflectivity values of the EZ and ELM, implying that more extensive fluid collections induce more profound damage to these layers.^13^

Moreover, Dursun and Dursun^16^ highlighted that outer nuclear layer thickness, which serves as an indirect marker of the health of photoreceptors, remains reduced even after the complete resolution of SRF in acute CSCR. This finding aligns with our results, which indicate that even after the SRF is managed, the reflectivity values in affected regions remain significantly lower than those in healthy controls. The implication here is that the damage caused by SRF is not merely transient but can lead to prolonged, possibly irreversible alterations in retinal structure.^16^

### The Role of PED in Disease Progression

In addition to SRF, the presence of PED has a significant impact on outer retinal integrity. In this study, PED was associated with significantly lower reflectivity of the nasal EZ compared to patients without PED. This observation suggests that PED may exacerbate the adverse effects of SRF on retinal structures. Xu et al.^17^ found that PED is influenced by increased choroidal blood flow, which correlates with SRF presence and increased choroidal vascular density in patients with CSCR. The increased vascular flow and PED are thought to contribute to the mechanical displacement of photoreceptors and the RPE, leading to further reduction in reflectivity and retinal integrity.^17^

Interestingly, PEDs have also been shown to contribute to the transition from acute to chronic CSCR. According to Huang et al.,^18^ biomarkers such as increased SRF and hyperreflective masses can predict the progression to chronic CSCR. The presence of PED, combined with larger SRF, suggests a more advanced stage of the disease, which might require different therapeutic approaches. Identifying patients with PED and monitoring their reflectivity changes could thus be an essential step in preventing the progression to chronic CSCR.^18^

Although reflectivity measurements in OCT imaging can be influenced by external factors such as signal strength, media clarity, and beam angle, our study design aimed to minimize these confounders. We applied a strict quality threshold, performed all imaging under mydriasis using a single device and operator, and employed a consistent protocol across all patients. More importantly, we calculated relative reflectivity indices by normalizing the EZ and ELM values to the RPE band, which is a widely accepted technique to reduce interindividual variability due to signal differences.^10^^–^^12^ The consistent and significant reduction in relative reflectivity values across multiple retinal layers and the layer-specific correlations with SRF parameters strongly support the biological validity of our findings and argue against technical artifacts as the primary source of variation.

### Clinical Implications for Reflectivity Monitoring and Treatment Strategies

The results of the present study highlight the necessity of periodic monitoring of reflectivity alterations in patients diagnosed with CSCR. As noted by Maltsev et al.,^14^ PROS thinning and other structural changes can serve as biomarkers for disease activity and treatment response. This mechanism suggests that incorporating quantitative analysis of retinal layer reflectivities into clinical practice could enhance the ability to personalize treatment plans for patients with CSCR. For instance, early intervention with therapies aimed at reducing SRF, such as mineralocorticoid receptor antagonists or photodynamic treatment, could preserve retinal layer integrity and improve visual outcomes.^14^

Moreover, photoreceptor integrity, as indicated by EZ reflectivity, can be used to evaluate treatment efficacy. Yu et al.^19^ demonstrated that the structural integrity of the EZ prior to photodynamic therapy could serve as a predictor of visual outcomes following treatment. In our study, we observed that decreased EZ reflectivity was correlated with larger SRF base widths and areas, indicating that the extent of fluid accumulation is a critical determinant of photoreceptor health. This information may prove helpful in determining the optimal timing for intervention in patients with significant EZ damage who may benefit from earlier or more aggressive treatment to mitigate further photoreceptor loss.^19^

### Insights Into Pathophysiology and Future Research Directions

The reflectivity changes observed in the current study offer insights into the pathophysiology of CSCR, particularly with regard to the mechanisms by which SRF and PED contribute to retinal damage. CSCR is characterized by disruptions in choroidal blood flow, leading to increased vascular permeability and leakage into the subretinal space. Studies by Chung et al.^20^ and Singh et al.^21^ have shown that choroidal thickness and abnormalities in the choriocapillaris are directly linked to disease severity and the risk of recurrences.^21^ The current study adds to this body of knowledge by showing that the extent of SRF and the presence of PED both significantly reduce the reflectivity of the outer retinal layers, suggesting ongoing damage and remodeling in the outer retina.

The main limitations of this study include its cross-sectional retrospective design and the relatively small sample size, particularly in the subgroup comparison between acute and chronic CSCR. This limited statistical power, and although several intergroup differences reached significance, the findings should be interpreted with caution. Nevertheless, further observational studies involving a larger number of patients are required to substantiate the findings of the present study. Another methodological limitation concerns the spatial resolution of our reflectivity analysis. Reflectivity was measured at three representative retinal points (central, nasal, and temporal) and averaged over short vertical segments to improve reproducibility and minimize local variability. While this approach does not capture the full spatial heterogeneity across the SRF area, it was selected as a practical and anatomically guided strategy that is technically feasible in clinical OCT data sets. More advanced approaches, such as continuous or boundary-guided sampling using polygonal or freehand region of interest, can be challenging to implement reliably, especially in the presence of outer retinal distortion. Although ImageJ is one of the most widely validated tools for OCT image analysis, accurately tracing irregular and undulating layers such as the ELM or EZ with these tools is technically challenging and may introduce inconsistency. Our approach is also consistent with several previously published studies.^6^^–^^12^

Although the groups differed in age, this was statistically adjusted using linear regression, and the control groups were age-matched. All participants were Caucasian, which minimizes potential variability due to ethnicity but limits the generalizability of our findings to other populations. Additionally, all OCT scans were acquired using a standardized protocol.^6^^–^^12^ A single experienced grader analyzed them, and minor variations in image acquisition angle may have subtly influenced reflectivity measurements. These factors are acknowledged as methodological limitations.

In conclusion, the quantitative findings of this study highlight the intricate relationship between retinal structure, SRF dimensions, and visual function in CSCR. The preservation of the outer retinal layers, particularly the EZ, is crucial for optimal visual outcomes, and timely intervention to reduce SRF is essential to prevent irreversible damage. The associations between reduced reflectivity and increased SRF dimensions underscore the need for regular, quantitative OCT assessments to guide personalized treatment approaches. As research continues to evolve, a deeper understanding of these relationships will enhance the management of CSCR, ultimately improving patient outcomes and reducing the likelihood of chronic visual impairment.
